# Knowledge, attitude and practice toward the mHealth app Mawid: a cross-sectional study

**DOI:** 10.1093/inthealth/ihac062

**Published:** 2022-09-15

**Authors:** Abeer Alharbi

**Affiliations:** Health Administration Department, Business Administration College, King Saud University, P.O.Box 145111, Riyad 11451, Saudi Arabia

**Keywords:** appointments, healthcare, mHealth, mobile apps, primary healthcare, Saudi Arabia

## Abstract

**Background:**

The Mawid app is a mobile appointment system that may improve access to primary healthcare services in Saudi Arabia. This study investigates the Saudis’ level of knowledge of the Mawid app, their attitude towards it and their practice or use of it.

**Methods:**

A cross-sectional design was used. The data were collected using an online survey via Google Forms from April to May 2021. Linear regression and binomial logit models were used to answer the research questions.

**Results:**

A total of 916 responses were collected. There were significant effects of gender, age, marital status, occupation, income and region on knowledge scores (p<0.05). Higher knowledge scores predict greater agreement that the Mawid app facilitates access to health services (p<0.001). The probability of using the app is predicted to be greater among individuals who agree that the Mawid app facilitates access to care (p<0.001). The probability of using the app is predicted to be higher among individuals who have a higher frequency of using primary health centres (p<0.001).

**Conclusions:**

The Ministry of Health has declared eHealth as a transformational enabler for patient-centric care. This study found that the Mawid app facilitated access and improved healthcare services. Knowledge positively influences attitudes toward the app and subsequently its use.

## Introduction

The Ministry of Health (MOH), through its network of hospitals and primary healthcare (PHC) centres, is the largest provider of healthcare services in Saudi Arabia, being responsible for >75% of the healthcare services, with the remaining 25% provided by private facilities.^1^ There are 2257 PHC centres that provide preventive and curative services, with 7.53 per 100 000 population.^1^ PHC services have improved considerably in the past 40 years, which has resulted in better health outcomes, including lower infant mortality rate, lower incidence of communicable diseases and an increase in the average life expectancy.^2^ At present, the Saudi population is estimated to be 21 million, 6% of whom are ≥65 y of age.^3^ It is predicted that the older population will increase and make up 18.4% of the total population by 2050.^4^ While the rate of communicable diseases has decreased, that of non-communicable diseases, especially cardiovascular diseases and diabetes, have increased.^5^ Aging, change in disease pattern and a rapid increase in population make access to healthcare services an important and urgent issue not only in Saudi Arabia, but also in other high- and low-/middle-income countries.^6–10^ Health expenditure as a share of gross domestic product for Saudi Arabia was 6.4% in 2020.^11,12^ The budget for healthcare in 2021 was SAR 175 billion (US$46.4 billion), an increase of 4.6% of the SAR 167 billion (US$44.2 billion) budgeted in 2020.^12^ The government spending as a percentage of total healthcare expenditures accounts for the majority of the total healthcare expenditures and stands at 75%, while the share of private sector expenditures stands at 25%.^1^ Healthcare costs are increasing in emerging markets across Asia, Latin America and Africa,^6–10^,^13^ and Saudi Arabia is facing these same increases.

However, despite large spending on healthcare, PHC services still face some challenges in terms accessibility,^2,14,15^ defined as the ease with which individuals get the needed care from their chosen physicians within a short time of making the chief complaint.^16^ The manner in which resources are organized to accommodate access, such as the appointment system through mobile health applications, can improve access to PHC services.^17^ A study in Saudi Arabia found that enabling patients to manage their appointments in the PHC system could help reduce the number of missed appointments.^18^ Also, the lack of an appointment system was found to be a barrier to accessing PHC services.^19^ Improved accessibility should therefore lead to better patient satisfaction, which in turn should improve the quality of primary healthcare.^20^

Healthcare systems worldwide are moving towards the adoption of advanced information and communication technologies as a means of improving the quality and accessibility of healthcare services.^21–23^ In Saudi Arabia, the MOH has declared that eHealth is a primary transformational enabler of high-quality and patient-centric care. eHealth efforts in Saudi Arabia have been found to be efficient in reducing the time, cost and the amount of effort required to provide care for patients.^24^

### Mawid app

**Figure 1.**
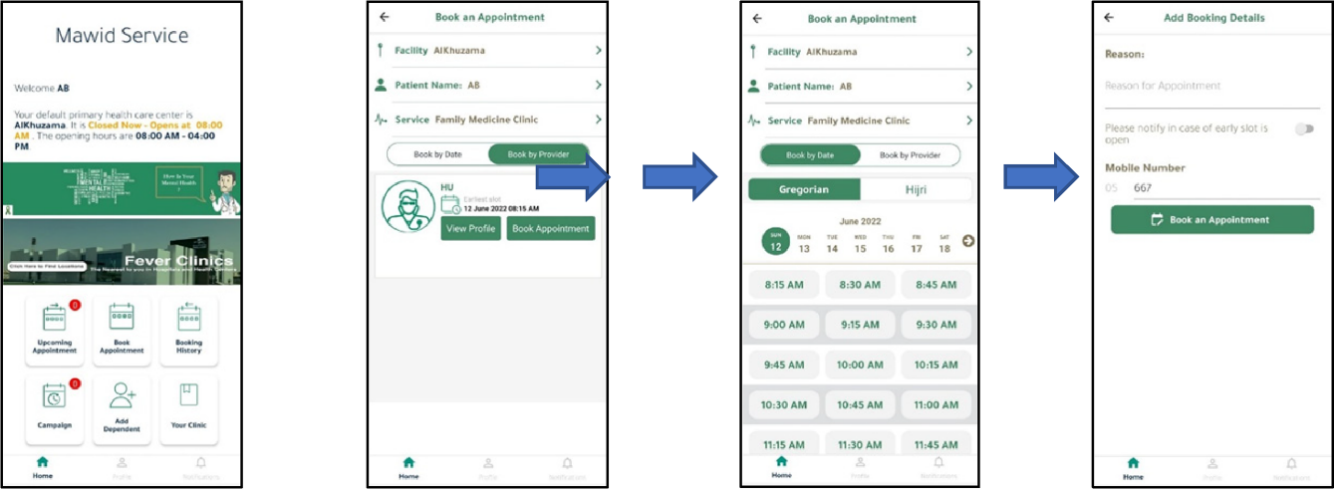
Mawid app interface.

In December 2017, the MOH launched the mHealth app Mawid as part of the eHealth strategies aimed to improve the patient experience. The Mawid app is a free mobile application that enables patients to book, cancel and/or reschedule their appointments at PHC centres, as well as manage their referral appointments to general and specialized hospitals. eReferrals were found to be successful in reducing wait times and improving access to secondary care as well as the information accuracy of referral patients.^25^ The Mawid app utilizes Google maps to help users find the healthcare centre nearest to them and to book appointments at >2000 healthcare centres across Saudi Arabia. The app allows users to know the number of available appointments in all healthcare centres as well as the actual number of outpatients, and this helps to increase accessibility and patient satisfaction. The app design is functional and easy to use. The app is available for both Android and iOS. Once the user has downloaded the app, they are required to sign in with their username and password. The user is taken to another window and is required to choose their default PHC centre on the app. They can change their default PHC centre at any time. The main screen shows icons for upcoming appointments, booking an appointment, previous appointments, add a dependent and information about the user's selected PHC centre, such as working hours, website and contact numbers. The app has other screens for personal information and notifications where the user gets reminders about upcoming appointments and referrals. For easy and functional use, the app design took into account the colour scheme, main page layout, buttons of the right sizes in the right places, balanced fonts and logical alignment, icons that help but never confuse and images that add value to the app purpose (Figure 1).

**Figure 2.**
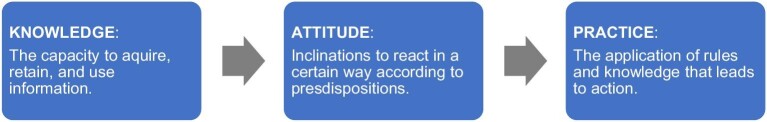
Relationship of KAP.

The development of the Mawid mobile app was driven by the expectation that the adoption and use of information and communication technologies would provide sustainable solutions and improve access and satisfaction with the PHC services in Saudi Arabia. The estimated monthly global downloads of the Mawid app is 46 367 on Google Play and 43 915 on Apple Store.^26^ Recent studies have shown that use of the Mawid app was lowest compared with other MOH mobile app initiatives^27^ and that the general public still lacked knowledge and experience with the app.^28^ The Knowledge, Attitude and Practice (KAP) theory divides the process of human behaviour change into three steps: acquiring knowledge, generating attitudes/beliefs and forming practices/behaviours. It states that during this process, human health behaviours can also be effectively changed (Figure 2).^29–31^

Therefore this study aimed to investigate the Saudis’ level of knowledge of the Mawid app, their attitudes towards it and their practice or use of it; to identify the demographic factors that may influence their level of knowledge about the Mawid app; and to explore how attitudes and usual source of care influences practices regarding the Mawid app.

The findings of this study could provide important aspects for policymakers to consider when designing awareness campaigns for the use of mHealth apps. Also, with the ever-increasing mobile market and adoption of mHealth around the world, this study could provide significant implications for similar health systems in the Middle East and beyond.

## Methods

### Design

This study applied a cross-sectional, quantitative design to identify the publics’ KAP when using the Mawid app in Saudi Arabia. This study design allowed for exploring the respondents’ KAP as well as an assessment of the relationship between the respondents’ knowledge and attitude with the practice of using the app. In addition, the design enabled the testing of whether there were differences in the respondents’ knowledge and practice across certain demographic variables.

### Population and sampling

The study population consisted of all those in Saudi Arabia ≥18 y of age. Based on the statistics of the General Authority of Statistics and the estimated percentage of smartphone users, the total study population was approximately 25.6 million users.^32^ The sample size was calculated using a margin of error of ±5%, a confidence error of 95%, a 50% response distribution and a population size of 21 million to arrive at the minimum required sample size of at least 385 participants. However, a convenience sampling method was used to collect the study data from the study population, of which 916 responded.^33^ Other factors that encouraged the researcher to use convenience sampling included its low cost and the quick and easy selection of individuals.^34^

### Survey questionnaire and data collection

An online questionnaire was developed using Google Forms and then distributed to the study population in April–May 2021. The questionnaire included two sections, a demographic section and a KAP section. The demographic data investigated the population characteristics of gender, age, marital status, place of residence, education, income and occupation. The KAP questions covered the domains of knowledge, attitude and practice. The knowledge domain was assessed using five questions focusing on the essential features and services provided by the Mawid app with three possible answers: true, false, I don't know. A correct answer was given a score of 1 and an incorrect or I don't know received a score of 0. The knowledge score was then calculated by summing up the correct answers. The attitude domain contained three statements expressing attitudes towards the Mawid app and other mHealth apps provided by the MOH. Respondents were asked to rate these statements on the 5-point Likert scale [1 (strongly disagree) to 5 (strongly agree)]. Finally, the practice domain assessed the respondents’ use of the Mawid app with a close-ended ‘yes/no’ question. Other practice domain items included the use of PHC centres, public health services and private health services, which were rated on a 5-point Likert scale [1 (never) to 5 (always)].

### Statistical analysis

The data were analysed using SPSS version 23.0 (IBM, Armonk, NY, USA). Range, mean and standard deviation (SD) were calculated for the KAP items. Frequencies and percentages were calculated for the demographic variables. A linear regression model was used to test the effect of each demographic variable on knowledge scores while holding all other demographic variables constant. Three linear regression models were used to test the effects of knowledge on each of the three attitude measures, while holding all demographic characteristics constant. For these models, responses to each of the attitude items were treated as numeric values ranging from −2 (strongly disagree) to 2 (strongly agree). Binomial logit models were used to test the effects of attitudes and usual source of care on the probability of using the app, while holding all demographic characteristics constant. A p-value <0.05 was considered to be statistically significant.

### Ethical considerations

Institutional review board (IRB) approval for the study was obtained from King Saud University (KSU; reference KSU-HE-21-795).

## Results

### Demographics

A total of 916 responses were collected for this study. As shown in Table 1, the majority of the sample was female (69.7%), <40 y of age (62.7%), married (65.5%), a public employee (40.4%), holding a bachelor's degree (58.3%), with an income of SAR 5000–20 000 (53.6%) and came mainly from the central region (59.3%). A detailed description of the demographics of the sample is shown in Table 1.

**Table 1.** Demographic characteristics of the sample (N=916)

Characteristics	n	%
Gender		
Male	278	30.3
Female	638	69.7
Age (years)		
18–29	300	32.8
30–39	274	29.9
40–49	192	21.0
≥50	150	16.4
Marital status		
Single	282	30.8
Married	600	65.5
Other	34	3.7
Place of residence		
Central	543	59.3
Eastern	243	26.5
Western	55	6.0
Southern	65	7.1
Northern	10	1.1
Education		
High school	296	32.3
Bachelor's	534	58.3
Master's	72	7.9
PhD	14	1.5
Income (SAR)		
<5000	363	39.6
5000–10 000	242	26.4
10 001–20 000	249	27.2
20 001–30 000	48	5.2
>30 000	14	1.5
Occupation		
Public employee	370	40.4
Private employee	268	29.3
Unemployed	278	30.3

### KAP

As shown in Table 2, 82% seemed to know that the Mawid app allowed individuals to book their appointments in PHC centres. However, most respondents (56%) mistakenly believed that the Mawid app allowed them to book their appointments in public (governmental) hospitals and 32% did not know if this was so. More responses to the knowledge aspect of the questionnaire are shown in Table 2.

**Table 2.** Responses to knowledge items (N=916)

	Knowledge item	Correct, %	Incorrect, %	Do not know, %
1	The (Mawid) App allows you to book your appointments in primary health centres	82	2	16
2	The (Mawid) App allows you to manage your appointments in the hospitals to which you are referred	53	9	38
3	The (Mawid) App allows adding and booking appointments for your dependents	73	2	25
4	The (Mawid) App allows you to book your appointments in government hospitals	12	56	32
5	The (Mawid) App allows you to book your appointments in private hospitals	36	15	49

As shown in Table 3, most of the respondents answered three of the five knowledge items correctly (median 3, mean 2.56 [SD 1.31]). The participants perceived the Mawid app as facilitating access to health services with a mean score (4.06 [SD 0.875]) higher than ‘agree’ on the Likert scale. They also recommended using the app for appointments (mean 4.19 [SD 0.879]) and perceived the MOH mHealth apps as contributing to the improvement of healthcare services. The Mawid app was used by 68% of the respondents for appointments. Respondents reported using PHC services with mean score (3.10 [SD 1.23]) slightly higher than ‘sometimes’ on the Likert scale, followed by using public health facilities (3.00 [SD 1.24]) and private health facilities (3.00 [SD 1.25]).

**Table 3.** Range, mean and SD of KAP (N=916)

Item	Range	Mean	SD
Knowledge score	0–5	2.56 (median=3)	1.31
Attitude			
The (Mawid) application facilitates access to health services	1–5	4.06	0.875
I recommend using the app for appointments	1–5	4.19	0.879
The MoH apps contribute to improving healthcare services	1–5	4.42	0.750
Practice			
I use the services of primary health centres (government)	1–5	3.10	1.23
I use public health facilities	1–5	3.00	1.24
I use private health facilities	1–5	3.00	1.25
I use Mawid app	0–1	0.68	0.466

### Impact of demographic variables on knowledge

A linear regression model was used to test the effect of each demographic variable on knowledge scores while holding all other demographic variables constant. Estimated coefficients for this model are shown in Table 4. This model shows significant effects of gender, age, marital status, occupation, income and region. Knowledge scores are predicted to be higher among males than among females when holding all other variables constant (p=0.006). When holding all other variables constant, respondents in the 18–29 y age group are predicted to have knowledge scores that are higher than those of the 40–49 y age group (p=0.02) and considerably higher than those of the 50–59 y age group (p<0.001). Single respondents are predicted to have lower knowledge scores than married respondents (p<0.001). Unemployed respondents are predicted to have knowledge scores that are lower than those of public employees (p<0.001) and lower than those of private employees (p=0.01). Higher-income individuals are predicted to have lower knowledge scores when holding all other characteristics constant. The highest knowledge scores are predicted to occur in the eastern region and the lowest in the northern region.

**Table 4.** Estimates of coefficients for a linear regression model predicting knowledge scores as the dependent variable

Variable	Coefficient	Standard error	t	p-Value
Female	−0.274	0.1	−2.737	0.006
Age 30–39 y	−0.026	0.137	−0.189	0.85
Age 40–49 y	−0.368	0.158	−2.328	0.02
Age 50–59 y	−0.701	0.179	−3.919	<0.001
Age ≥60 y	−0.366	0.321	−1.138	0.256
Single	−0.747	0.129	−5.808	<0.001
Divorced	−0.261	0.297	−0.879	0.379
Widow	0.355	0.333	1.065	0.287
Public employee	0.54	0.129	4.176	<0.001
Private employee	0.373	0.145	2.57	0.01
Bachelor's degree	−0.103	0.097	−1.062	0.289
Master's degree	−0.236	0.184	−1.28	0.201
PhD	0.369	0.371	0.993	0.321
Income SAR 5000–10 000	−0.325	0.127	−2.549	0.011
Income SAR 10 000–20 000	−0.173	0.145	−1.193	0.233
Income SAR 20 000–30 000	−0.553	0.232	−2.379	0.018
Income >SAR 30 000	−0.728	0.35	−2.077	0.038
Eastern	0.307	0.106	2.897	0.004
Western	−0.131	0.18	−0.73	0.466
Southern	0.114	0.171	0.664	0.507
Northern	−1.087	0.399	−2.723	0.007
Constant	3.175	0.312	10.172	<0.001

### Association between knowledge and attitude

Three linear regression models were used to test the effects of knowledge on each of the attitude measures while holding all demographic characteristics constant. For these models, responses to each of the attitude items were treated as numeric values ranging from −2 (strongly disagree) to 2 (strongly agree). Estimated coefficients for these models are shown in Table 5. Higher knowledge scores predict greater agreement that the Mawid app facilitates access to health services (p<0.001), greater agreement that the respondent recommends using the app (p<0.001) and greater agreement that MOH apps improve the level of healthcare services (p<0.001).

**Table 5.** Estimates from three linear regression models estimating the effect of knowledge scores on attitude variables

Variables	Coefficient	Standard error	t	p-Value
Mawid app facilitates access to healthcare	0.25	0.022	11.386	<0.001
I recommend using the app	0.277	0.022	12.881	<0.001
MOH apps improve the level of services	0.162	0.019	8.372	<0.001

Each model includes all the demographic characteristics as control variables, but the coefficients for the demographic variables are omitted from the table to save space.

### Association between attitude and practice

A binomial logit model was used to test the effects of attitudes on the probability of using the app, while holding all demographic characteristics and knowledge scores constant. As shown in Table 6, the probability of using the app is predicted to be greater among individuals who agree that the Mawid app facilitates access to care (p=0.027) and greater among individuals who agree that MOH apps improve the level of healthcare (p<0.001).

**Table 6.** Estimated coefficients for a binomial logit model estimating the effect of attitude on the probability of using the app

Variables	Coefficient	Standard error	t	p
Attitude: Mawid facilitates access to health services	0.335	0.151	2.218	0.027
Attitude: MOH apps improve healthcare services	0.679	0.148	4.57	<0.001

The model includes all the demographic characteristics and knowledge score as control variables, but the coefficients are omitted from the table to save space.

### Association between usual source of care and practice

A binomial logit model was used to test the association between the probability of using the app and the frequency of using each source of care, while holding constant the frequency of use of the other sources of care and all demographic characteristics [Table 7]. For this model, responses to the questions about how frequently the respondent uses each source of care were treated as numeric values ranging from 0 (never) to 4 (always). The model shows that the probability of using the app is predicted to be higher among individuals who have a higher frequency of using PHC centres (p<0.01). However, the probability of using the app among individuals who have a higher frequency of using private health services and public health services was not statistically significant (p>0.05).

**Table 7.** Estimated coefficients for a binomial logit model estimating the effect of usual source of care on the probability of using the app

Variables	Coefficient	Standard error	t	p-Value
Frequency of using primary health centres (government)	0.962	0.098	9.84	<0.001
Frequency of using public health services	−0.115	0.09	−1.278	0.201
Frequency of using private health services	−0.137	0.072	−1.897	0.058

The model includes all the demographic characteristics as control variables, but the coefficients for the demographic variables are omitted from the table to save space.

## Discussion

Healthcare systems worldwide, and particularly those in developing countries, are faced with challenges such as increased costs, aging, changes in disease patterns and a rapid increase in population,^6–10^ and Saudi Arabia is no different. One of the major approaches for improving the delivery of healthcare in Saudi Arabia is the adoption of digital healthcare. As the provision of healthcare through traditional methods continues to fall behind in terms of access and the quality of care, the introduction of technology and the use of mobile apps could be ways to overcome this deficiency. In doing so, the MOH has adopted mHealth to improve access to healthcare services, including PHC. Because appointment systems through mobile health applications can improve access to PHC services,^17^ the Mawid app, which acts as a central appointment system, has the potential to improve access to these services. This study examined the level of KAP toward the Mawid app among the Saudi population. It also identified demographic factors that may influence the level of knowledge about the Mawid app as well as explored how knowledge may affect attitudes towards it. Finally, it explored how practice was influenced by people's attitudes towards the Mawid app and other MOH mHealth apps and the usual source of care.

Most of the study respondents appeared to have sufficient knowledge about the Mawid app and seemed to know that it allowed them to book their appointments in primary health centres as well as to manage their referrals at general or specialized hospitals. However, most respondents mistakenly believed that the Mawid app allowed them to book their appointments directly in public or private hospitals, which was not an available feature. Ignorance of this fact might probably arise because, although PHC is the first level of the healthcare service, most Saudi citizens are accustomed to seeking care for non-urgent conditions through outpatient settings or in hospital emergency rooms.^35^ Their knowledge about the app seems to be influenced by demographic factors such as age, where younger persons had more knowledge about the mobile app than older persons. This finding is consistent with previous research that found younger individuals are more familiar with health mobile apps than the elderly.^36,37^ This is probably because these younger individuals are more technology savvy than elderly ones. This finding would seem to suggest the importance and impact of educating the older population on the use and advantages of mobile apps to improve their overall experience with healthcare services. In addition, the results show that married individuals have more knowledge about the Mawid app than single ones. A previous study found most PHC users were married.^38^ The fact that most people seek PHC services for child immunization and maternal and child healthcare^39,40^ could explain why married people were more knowledgeable about the PHC appointment app. Additionally, the study showed that people who were unemployed had lower knowledge about the Mawid app than public and private employees. This is consistent with a previous study that found Saudi public employees used more MOH mHealth apps than the unemployed.^37^ In addition, the study showed that male participants had more knowledge about the Mawid app than females, which was consistent with previous studies that observed males had a higher level of mHealth adoption than females.^37,41^ This difference between males and females in knowledge about the app should be recognized by decision-makers involved in the development of these apps to develop strategies to overcome this gender divergence. Interestingly, the participants who lived in the eastern region of Saudi Arabia had the highest knowledge level about the Mawid app. This is probably because the eastern region has the highest ratio of PHC centres to residents (1 PHC centre for every 21 250 residents),^1,3^ which may encourage the adoption of mHealth to improve access to PHC services. The study also revealed that there were differences in the level of knowledge between income groups. Higher-income individuals had the lowest level of knowledge. This is probably because high-income individuals prefer to seek healthcare services from the private sector.^42,43^

The study revealed that people had a positive attitude toward the Mawid app and other MOH mHealth apps. They believed that the MOH mHealth apps contributed to the improvement of healthcare services. They also believed that the Mawid app facilitated access to health services and they recommended using the app for appointments. These results are consistent with previous studies that found the Saudi population satisfied with the Mawid app and mHealth apps provided by the MOH.^27,37,44,45^ The attitudes towards the Mawid app seem to be influenced by their level of knowledge about the app. The more people knew about the app's availability and features, the greater they would perceive the Mawid app as facilitating access to care. This is consistent with the notion that when people acquire information about something, this tends to lead to the development of a predisposition to respond (an attitude), which in turn leads to behaviour (practice) that is in agreement with the attitude.^29,30^ Indeed, the study has revealed that the app's use is positively influenced by the users’ attitudes towards the MOH mHealth and Mawid apps. People who believed that MOH mHealth apps improved the delivery of care and that the Mawid app facilitated access to healthcare were more likely to use the app. In addition, the type of usual source of care seems to have an influence on app use, as persons who frequently used primary care centres were more likely to use the Mawid app. In contrast, the use of private and public healthcare facilities had no effect on the use of the Mawid app. This was expected, as the Mawid app only provides appointments for PHC centres and subsequently manages referrals to general or specialized hospitals.

The MOH used many methods for Mawid app distribution. The app was announced through the MOH website, social media and the internet. A recent study conducted to assess how MOH app users came to know about these apps found that social media networking (46.5%) was the most stated method. More than 30% of the respondents had heard or knew about MOH mHealth apps through the MOH website and accounts. Other methods of awareness included relatives and friends, the internet and other sources, which fell below 11%.^46^ Therefore, in order to expand knowledge of the app, which would ultimately improve attitude and practice, it is necessary to focus on the channels that are most likely would reach a large number of people such as social media.

As the adoption of mHealth is rapidly growing around the world, this study could provide important information for similar health systems in the Middle East and other developing countries. In the Middle East, the growing health issues and the high use of mobile devices made the area ripe for the use of mHealth to help improve the health of its population.^47^ For example, in the United Arab Emirates (UAE), mHealth solutions were part of the country's support for the Smart Cities Approach, as a mobile appointment system was developed to connect a huge number of hospitals and clinics with users throughout the UAE, enable people to look for doctors in different locations and make appointments that suit them.^48^ In the developing world, mHealth solutions have enabled many people to access high-quality healthcare. In Pakistan, a mobile appointment app has proved useful in making appointments with doctors and resolved many of the problems that patients faced in making an appointment.^49^ In rural Africa, mHealth is used to improve access to health services for patients who live long distances from PHC centres.^50,51^ According to existing literature, barriers contributing to the low adoption of mHealth in developing countries include inadequate health literacy, lack of effectiveness, safety, privacy, awareness and poor integration with the traditional healthcare system.^52^ The current study provides evidence that knowledge about mHealth apps increases positive attitudes towards these apps and eventually improves utilization. Thus more research is recommended to understand population awareness and attitudes toward these apps to approach this issue from a different perspective.

This study has some limitations. A convenience sample was used, which may not be fully representative of the study population. For example, there were more females in the study sample than males, while in the national population the ratio is 1.34 males to 1 female.^53^ Also, in the sample, the age group 18–29 y represents 32.8%, while in the national population it is 22.2%. The majority of the sample respondents reside in central region (59.3%), while in the national population 27.5% reside in the central region and 28.7% live in the western region. The majority of the sample respondents have a bachelor’s degree (58.3%), while in the national population, 66% have a high school degree and 28.1% have a bachelor’s degree or above. The majority of the sample (40.4%) work in the public sector, while in the national population, more people work in the private sector (55%).^53^ Thus we could not generalize the findings from the inferential statistics to the whole population. Therefore there is a need for future studies to be done with a more representative sample of the population in terms of gender, region, education, income and age, among other factors.

## Conclusions

In Saudi Arabia, the MOH has declared that eHealth is a primary transformational enabler of high-quality and patient-centric care. This study found that the mHealth Mawid app facilitates access and improves healthcare services for its users. The study respondents had sufficient knowledge about the app, showed positive attitudes towards the MOH mHealth and Mawid apps and frequently used the Mawid app to make their PHC appointments. This study provided evidence that knowledge positively influences the participants’ attitudes toward the Mawid app and, subsequently, its use by them. Gender, age, marital status, occupation, income and place of residence were significantly associated with their level of knowledge of the app. The frequent use of PHC was associated with a higher use of the Mawid app. These finding have provided some important factors for policymakers to consider when designing awareness campaigns for the use of the mHealth apps.

## Authors’ contributions

A.A. contributed to the research conceptualization, methodology, formal analysis and writing, review and editing of the manuscript.

## Data Availability

Data are available upon request.
